# Comparison of discriminant methods and deep learning analysis in plant taxonomy: a case study of *Elatine*

**DOI:** 10.1038/s41598-022-24660-1

**Published:** 2022-11-28

**Authors:** Andrzej Łysko, Agnieszka Popiela, Paweł Forczmański, Attila Molnár V., Balázs András Lukács, Zoltán Barta, Witold Maćków, Grzegorz J. Wolski

**Affiliations:** 1 Faculty of Computer Science and Information Technology, West Pomeranian University of Technology in Szczecin, Szczecin, Poland; 2 Institute of Biology, University of Szczecin, Szczecin, Poland; 3 Department of Botany, University of Debrecen, Debrecen, Hungary; 4 ELKH-DE Conservation Biology Research Group, Debrecen, Hungary; 5 Wetland Ecology Research Group, Centre of Ecological Research, Debrecen, Hungary; 6 ELKH-DE Behavioural Ecology Research Group, Department of Evolutionary Zoology and Human Biology, University of Debrecen, Debrecen, Hungary; 7 Department of Geobotany and Plant Ecology, Faculty of Biology and Environmental Protection, University of Łódź, ul. Banacha 12/16, 90-237 Łódź, Poland

**Keywords:** Plant sciences, Biological techniques

## Abstract

*Elatine* is a genus in which, flower and seed characteristics are the most important diagnostic features; i.e. seed shape and the structure of its cover found to be the most reliable identification character. We used a combination of classic discriminant methods by combining with deep learning techniques to analyze seed morphometric data within 28 populations of six *Elatine* species from 11 countries throughout the Northern Hemisphere to compare the obtained results and then check their taxonomic classification. Our findings indicate that among the discriminant methods, Quadratic Discriminant Analysis (QDA) had the highest percentage of correct matching (mean fit—91.23%); only the deep machine learning method based on Convolutional Neural Network (CNN) was characterized by a higher match (mean fit—93.40%). The QDA method recognized the seeds of *E*. *brochonii* and *E*. *orthosperma* with 99% accuracy, and the CNN method with 100%. Other taxa, such as *E*. *alsinastrum*, *E*. *trianda*, *E*. *californica* and *E*. *hungarica* were matched with an accuracy of at least 95% (CNN). Our results indicate that the CNN obtains remarkably more accurate classifications than classic discriminant methods, and better recognizes the entire taxa pool analyzed. The least recognized species are *E*. *macropoda* and *E*. *hexandra* (88% and 78% match).

## Introduction

The *Elatine* L. genus consists of cc. 15–25 ephemeral, aquatic species^1^, many of them considered as rare and threatened within their range. In recent years, several studies have been carried out on many of the species of the genus to clarify their distribution, ecology, molecular taxonomy, biology, cardiology and phenotypic plasticity^2–10^. These studies attribute the observed environmental induced phenotypic plasticity of the European species, since all the species has distinctive aquatic and terrestrial forms. These morphological variations of the species have led to taxonomical errors, since aquatic and terrestrial forms often described as separate species, and also made species identification difficult. Consequently, many authors have emphasized the importance of seed morphology in *Elatine* taxonomy, especially their shape, i.e. the degree of their curvature, and the structure of the seed coat^2,11–15^.

Recent studies on the taxonomy, phytogeography and morphology of the genus *Elatine* evaluated the morphometric features of seeds of 10 species (including all native European taxa) and revealed that, apart from the generative traits, only the seed morphology is valuable for taxonomic purposes^6,9,16^. However, it is not obvious how to measure and characterize seed shape or seed coat, and whether the analyzed traits are sufficient to distinguish species. Measurements conducted included seed width and height morphometry, seed bend, size and number of pits. This work was conducted manually using morphometric measurement software, which requires a great deal of care and time commitment. These results also, depending on the identifying individual, may not be very reproducible (which can affect the result), unlike automatic determinations conducted by computer, which are always the same on the same images.

To avoid subjective approaches that often resulted in misinterpretations of a species character, scientist apply comparative methods requires measured data of species traits, that allow us to classify species in a statistical basis. For such classification purposes, various types of discriminant analysis are often used including Linear Discriminant Analysis (LDA) and the related Fisher's Linear Discriminant Analyses (FLD)^17,18^, Quadratic Discriminant Analysis (QDA, Partial Least Squares Discriminant Analysis, Regularized Discriminant Analysis^19^, and the K-nearest neighbor and tree classification method^20^. There are also methods, such as the neural networks, less used in taxonomy^21^, but which now becoming very popular techniques in industry^22–24^ and in pharmaceutical science^25^. Moreover, their application in classical botanical, zoological or ecological purposes also recognised as very useful cognitive tool^26,27^. Their primary advantage is their comparable precision to human determinations and the high reproducibility of the results obtained.

To date, taxonomic research has rarely employed neural networks, and no attempts have been made to compare the obtained identifications with more classical discriminant methods. On the other hand as the literature review shows, these modern methods of analysis provide a new, significantly different view of the issues under study. Thus, in taxonomic studies, we may obtain different results from those currently recognized in the literature. This has become the reason to take up this innovative and novel research topic. The aim of the present study was to evaluate the potential of these new methods in taxonomic research. To this end, the study has the following goals: the construction of an algorithm for identifying taxa of the genus *Elatine* on the basis of seed characteristics, an analysis of the obtained metric data using deep learning methods, a comparison of these findings with the results of classical morphometric methods, and final confirmation of the obtained results in terms taxonomic classification.

## Materials and methods

### Seed material

**Figure 1:**
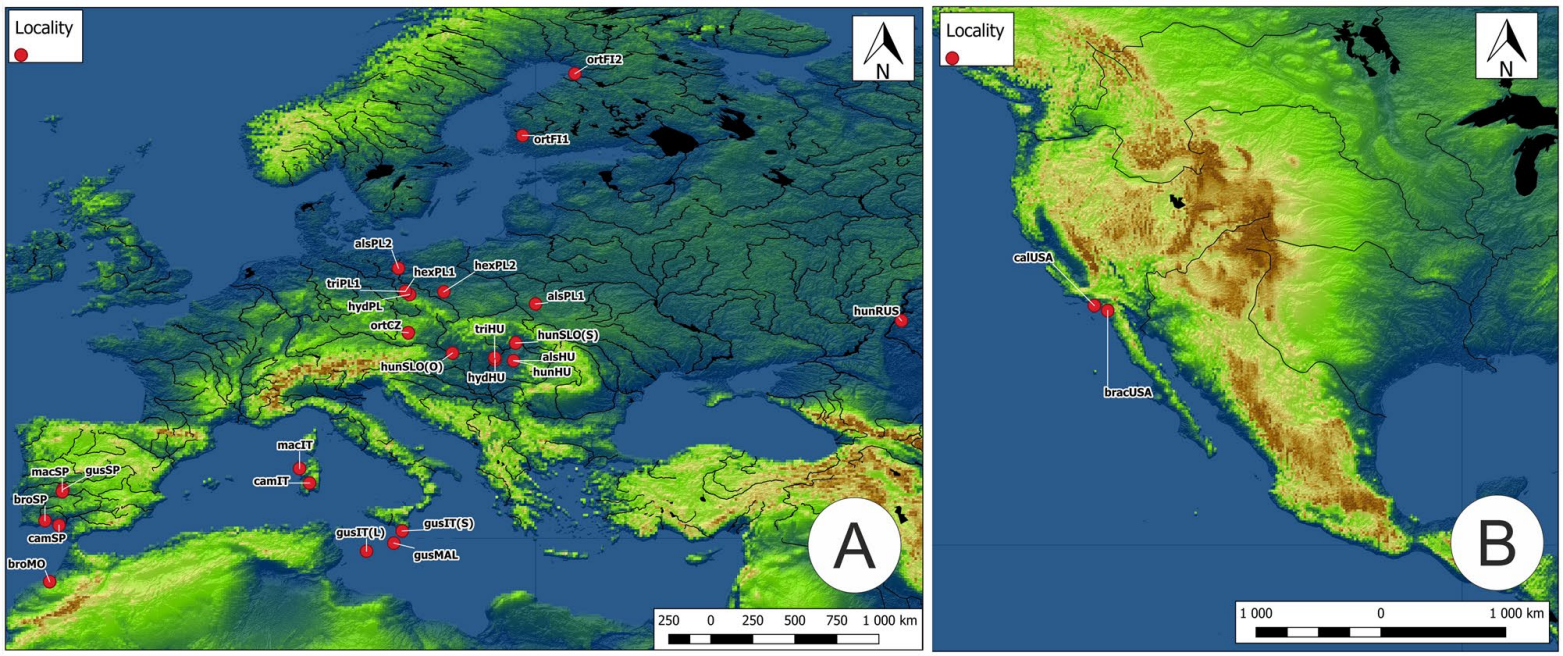
Location of the tested seed populations of *Elatine*; (**A**) Europe and Africa, (**B**) North America; explanation of abbreviations in Table 1.

In total, seeds from 12 taxa of the genus *Elatine* were collected. Some of the seeds was collected in the field, others originated from cultivated plants or from herbarium specimens stored in Herbarium of Debrecen (DE) and Herbarium Stetinense (SZUB). Seeds were originated from two or three populations of a species; but, *E*. *californica* and *E*. *brachysperma* were only obtained from single populations. As such, 28 populations from 11 countries in Europe, Africa and North America were used for the study (Fig. 1). The distance between the populations of each species ranged from about 10 to 2000 km (Table 1).

**Table 1:** Species included in the study, origins of the studied populations, information voucher specimens, and the number of seeds.

No	Acronym	Name	Origin	Latitude	Longitude	Collector, voucher	No. of pictures of seeds
1	alsHU	*Elatine alsinastrum* L	Hungary: Konyár**	47.31	21.67	*Molnár V.A* DE-2226	50
2	alsPL1	*E. alsinastrum*	Poland: Staw Noakowski*	50.80	23.03	*Popiela A* SZUB 008,756	51
3	alsPL2	*E. alsinastrum*	Poland: Strzelczyn	53.01	14.54	*Popiela A* SZUB 015,968	45
4	bracUSA	*E. brachysperma* A. Gray	USA: Fallbrook	33.46	− 117.37	Löki V	50
5	broMO	*E. brochonii* Clavaud	Morocco: Ben Slimane**	33.62	− 7.07	*Lukács B.A* DE-43230	50
6	broSP	*E. brochonii*	Spain: San Silvestre de Guzmán**	37.4	− 7.36	*Molnár V.A* DE-37684	50
7	calUSA	*E. californica* A. Gray	USA: Los Angeles	33.82	− 118.34	Löki V	50
8	camIT	*E. campylosperma* Seub	Italy: Sardegna, Gesturi**	37.12	− 6.49	*Molnár V.A* DE-37423	50
9	camSP	*E. campylosperma*	Spain: El Rocio, Donana**	39.73	9.03	*Molnár V.A* DE-37681	55
10	gusIT(L)	*E. gussonei* (Sommier) Brullo, Lanfr., Pavone & Ronsisv	Italy: Lampedusa	35.51	12.56	Molnár V.A. & Lukács B.A	50
11	gusIT(S)	*E. gussonei*	Italy: Sicily, Modica**	36.76	14.77	*Molnár V.A* DE-38750	50
12	gusMAL	*E. gussonei*	Malta: Gózó: Ta' Sannat**	36.01	14.25	*Molnár V.A* *& Lukács B.A* DE-43229	50
13	gusSP	*E. gussonei*	Spain: Casar de Cáceres**	39.33	− 6.25	*Molnár V.A* DE-43231	50
14	hexPL1	*E. hexandra* (Lapierre) DC	Poland: Janików	51.57	14.96	*Popiela A* SZUB 015,964	33
15	hexPL2	*E. hexandra*	Poland: Milicz*	51.55	17.35	*Popiela A* SZUB: 010,851	50
16	hunHU	*E. hungarica* Moesz	Hungary: Konyár**	47.31	21.67	*Molnár V.A* DE-22266	50
17	hunRUS	*E. hungarica*	Russia: Volgograd**	49.76	45.7	*Mesterházy A* DE-37484	50
18	hunSLO(O)	*E. hungarica*	Slovakia: Okánikowo	47.78	17.88	*Eliáš P* SZUB 010,523	25
19	hunSLO(S)	*E. hungarica*	Slovakia: Somotor	48.40	21.80	*Eliáš P* SZUB ?	24
20	hydHU	*E. hydropiper* L	Hungary: Tiszagyenda**	47.36	20.52	*Molnár V.A* DE-22273	50
21	hydPL	*E. hydropiper*	Poland: Parowa	51.38	15.23	*Popiela A*	50
22	macIT	*E. macropoda* Guss	Italy: Sardegna: Olmedo**	40.63	8.41	*Molnár V.A* DE-37424	50
23	macSP	*E. macropoda*	Spain: Casar de Cáceres**	39.19	− 6.29	*Molnár V.A* DE-37692	50
24	ortCZ	*E. orthosperma* Düben	Czech Republic: Klášter*	49.02	15.15	*Šumberova K*	50
25	ortFI1	*E. orthosperm*	Finland: Kokemäki	61.23	22.23	*Suominen J* H 439,800	25
26	ortFI2	*E. orthosperma*	Finland: Oulu*	65.06	25.47	*Mesterházy A* DE-43232	50
27	triHU	*E. triandra* Schkuhr	Hungary: Kisköre*	47.50	20.50	*Molnár V.A* DE-22282	41
28	triPL1	*E. triandra*	Poland: Janików	51.57	14.96	*Popiela A* SZUB 010,520	50

*Cultivation in Poland.

**Cultivation in Hungary.

*Elatine hungarica*, *E*. *hydropiper* and *E*. *triandra* are protected species and were sampled in Hungary with the permission of the Hortobágy National Park Directorate (Permission id.: 45-2/2000, 250-2/2001). In the case of other species only seeds were collected in the field, not specimens, namely permission is not required. Plant breeding was carried out in growing chambers. In Poland and Hungary, a *Elatine* sp. growing permit is not required. Attila V. Molnar and Agnieszka Popiela were responsible for the formal identification of the plant material used in this study. All methods were carried out in accordance with relevant guidelines in the method section.

High-resolution scanning electron microscope (SEM) images of seeds were taken from several individuals from each population (24 to 50 seed photos perpopulation of the species) (Table 1). In total, 1299 SEM images of the seeds were obtained at × 200 magnification using a Zeiss Evo SEM.

### Numerical analysis

#### Deep convolutional networks (CNN): based seed classification

Our approach is based on a so called deep learning, a subdomain of machine learning. The base algorithm uses convolutional neural network that is biologically-inspired algorithm for data processing^28^. CNNs are usually devoted to solving visual tasks, where large training datasets are available yet no clear and deterministic solutions can be employed. CNNs are a direct extension of well-known artificial neural networks (eg. Multi-layer Perceptron), proposed in the second half of twentieth century^29^.

The input data were provided as raster images of 1024 × 768 pixels, represented by 8-bit gray-scale values. The data was taken directly from the electron microscope; each image includes not only the image data of the seed, but various metadata related to the imaging parameters, date and time of collecting etc. In many cases, the seed in the image is accompanied by fragments of other seeds, miscellaneous additional objects and various artifacts (Supporting Information Fig. 1).

#### Processing algorithm overview

The processing algorithm consists of two main steps. Due to the small number of images, it is not possible to employ a simple, yet effective, end-to-end learning approach. Instead, a two-tier deep-learning approach was used. The first tier contains a CNN (Convolutional Neural Network) devoted to image pre-processing, and the second tier a CNN responsible for feature extraction and classification. The input to the first stage is the image collected from the electron microscope, while the output is a standardized image with a fixed size. The second tier takes this input and classifies it as belonging to one of 12 classes.

#### Initial preprocessing

The preprocessing stage consisted of four steps: 1. seed segmentation; 2. image cropping; 3. image padding; 4. image scaling to a final resolution. Seed segmentation was performed using a U^2^-Net approach^30^ as implemented in the rembg library^31^. The CNN was not trained with our set of seed images: it was used “as is”. In a significant majority of cases, it was found to segment the seeds with high accuracy (average 93.4%; median 93.5%).

During this process (Supporting Information Fig. 2) small objects and text areas were removed successfully; however, in some cases, slightly larger objects were present in the image, which were considered important by the U^2^-Net. In such cases, additional post-processing was performed to remove any unnecessary objects (Supporting Information Fig. 2B). In the case of background contamination present, it was important that the seeds did not come into contact with the analyzed image. In cases where the image could not be separated, it was not considered.

**Figure 2:**
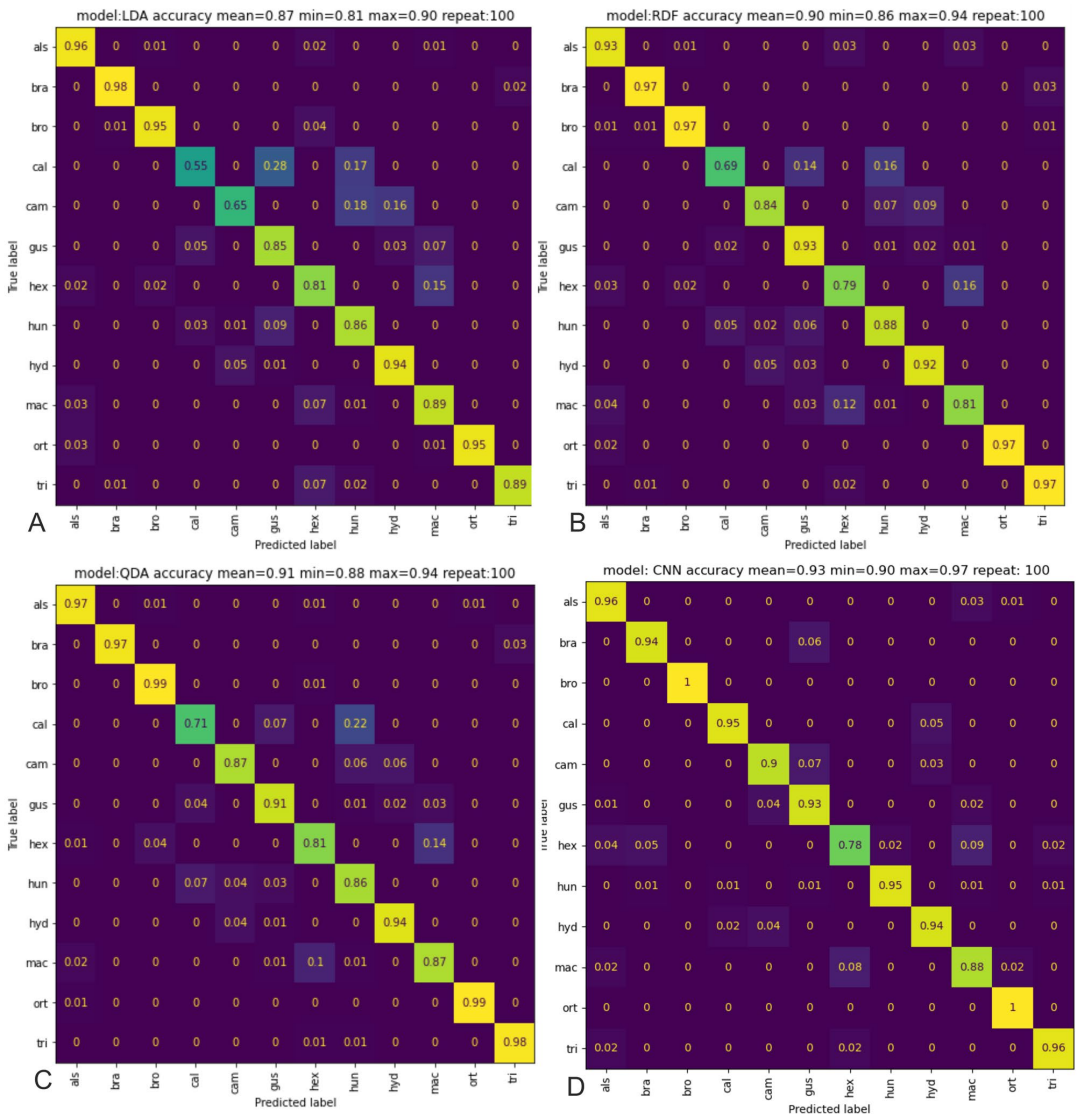
Confusion matrix in 100 repetitions in methods: (**A**) LDA; (**B**) RDF; (**C**) QDA; (**D**) CNN analysis.

The segmented image is then subjected to classical computer vision processing, namely thresholding, contour detection and removal of all contours but the most prominent one. Thus the main object in the scene, i.e. the analysed seed, was isolated (Supporting Information Fig. 3). This method has been validated in several previous studies^32,33^.

**Figure 3:**
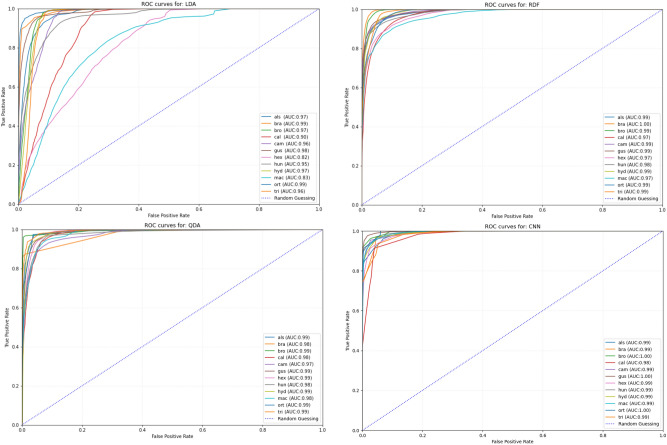
ROC curve and AUC value, for 100 random samples of statistical analysis: Random Forest (RDF), Linear Discriminant Analysis (LDA), Quadratic Discriminat Analysis (QDA) and Deep Learning CNN. (als)—*Elatine alsinastrum* L.; (bra)—*E. brachysperma* A. Gray; (bro)—*E. brochonii* Clavaud; (cal)—*E. californica* A. Gray; (cam)—*E. campylosperma* Seub; (gus)—*E. gussonei* (Sommier) Brullo, Lanfr., Pavone & Ronsisv.; (hex)—*E. hexandra* (Lapierre) DC.; (hun)—*E. hungarica* Moesz; (hyd)—*E. hydropiper* L.; (mac)—*E. macropoda* Guss.; (ort)—*E. orthosperma* Düben; (tri)—*E. triandra* Schkuhr.

Following this, a binary mask was used to extract the area contains occupies the seed and crop it to a square of a consistent size. The cropping was combined with padding, as many seeds are rectangular in shape and simple cropping would remove necessary image parts. In our case the output cropped/padded image measured 128 × 128 pixels (Supporting Information Fig. 4).

#### Data augmentation

Since the number of original samples is low, data augmentation was needed to train a robust classifier based on the CNN; therefore, resultant images were used in the classifier. In this case, the following classical image processing operations were used: 1. changing brightness and contrast of input images (five variants of the original image); 2. sharpening images using convolution filtering (six variants of the original image, combined with flipping); 3. blurring images using convolution filtering (12 variants of the original image, combined with flipping); 4. rotating images (61 variants of the original image, cyclic rotation by 15 degrees combined with flipping); 5. Simple image flipping along horizontal and vertical axes (2 variants) (exemplary augmentation results are presented in Supporting Information Fig. 5).

#### Training the model

The input to the net is a 128 × 128 pixels, single channel, gray-scale image. The output consists of a one-hot-vector encoding 12 classes related to the seed species investigated. It was found that 128 × 128 was the minimal size that could capture all the important visual characteristics of the seed: smaller images (32 × 32 and 64 × 64) did not manage to describe the seed with sufficient precision, while larger images (256 × 256) entailed greater memory use and computational effort without significantly increasing accuracy.

At the feature extraction stage, the net consists of four convolutional layers (16, 32, 64 and 128 filters with kernels equal to 3 × 3, respectively), followed by batch normalization and max pooling layers (with kernel equal to 2 × 2). The activation function in all these cases is ReLU. The classification is performed using further two fully-connected dense layers followed by dropout layers. The output consists of a dense layer of 12 neurons activated with the SoftMax function (The net structure is presented in Supporting Information Fig. 6).

In total, the net needed to be trained for 168,076 parameters, which makes it rather lightweight and quite easy to train. The training was performed using Adam optimizer with a learning rate equal to 1e−4 and decay equal to 1e−4. The system used binary crossentropy as a loss function to control the training progress. The training was set to a maximal number of 4500 epochs with an early stopping rule to break the process, when the training reaches the plateau. Training/validation sets were created by randomly splitting the original in 75%/25% proportions. At the training stage, 974 original images yielded 83764 augmented images (86 variants per one image). The testing employed 325 original images (without augmentation).

### Morphometric analyses

The tested material was subjected to morphometric measurements to allow statistical analysis. A total of six parameters were measured: object area; profile-specific perimeter (object perimeter); object rectangle a (length); object rectangle b (width); angle, curvature, number of pits on the seed coat (Supporting Information Fig. 7).

Seed classification was performed on the basis of the above six variables using three different statistical methods: Linear Discriminant Analysis (LDA); Quadratic Discriminant Analysis (QDA) and Random Decision Forest (RDF)^17,34,35^. The first two methods (LDA and QDA) are classic classifiers that use LDA; these can be used for supervised dimensionality reduction by projecting input data onto a linear subspace consisting of directions that maximize the separation between classes. This method is only appropriate for multiclass data sets.

Quadratic Discriminant Analysis (QDA) can be employed when the covariances differ, while, Random Decision Forest (RDF) is a machine learning method based on decision trees used for *inter alia* classification and regression. The trees are built in such a way that each one depends on the value of an independently-sampled random vector.

The Python scikit-learn libraries and modules in Python version 3.8 allow LDA, QDA and RDF analysis. Each analysis was repeated in a loop a hundred times, with all statistical results removed after each loop and recalculated in the next loop. On this basis, the average correctness of all analyses was calculated. The number of decision trees in the RDF (n estimators) method was set to 100. In each repetition, the data was randomized a new.

A module sklearn.metrics.confusion_matrix was used to assess the correctness of prediction in all methods. The importance of the individual variables in the data sets and individual classification methods was assessed using the module sklearn.inspection.permutation.importance (which calculates the importance of each variable in the analysis). The selection for the training and test groups was carried out using the module sklearn.model_selection.train_test_split. The data set was divided for each of the 12 taxa, with a size of 75% (training set) and 25% (test set), respectively, in such a way that the training and test set cases did not coincide in any of them. In each repetition, the data was randomized a new.

The map (Fig. 1) was made by the first author of the article (A.Ł.), based on open data: Space Shuttle Radar Topography Mission (SRTM) made available by https://earthexplorer.usgs.gov/. The map is the result of the transformation of the source data. The map was made in QuantumGis software (QGIS 3.16) (https://www.qgis.org). A list of the software used here is available in the first author of this manuscript.

## Results

### General results

Among the selected discriminant methods, the Quadratic Discriminant Analysis (QDA) showed the highest percentage (91.23% on average) of correct fit for the test set following 100 repetitions; this was followed by RDF (89.96%) and LDA (86.78%). However, deep machine learning based on CNN (Convolutional Neural Network) was more accurate, with an average fit of 93.4% after 100 repetitions (Table 2; Supporting Information Fig. 8).

**Table 2:** The results of the individual analysis types.

Model	Min (%)	Max (%)	Mean (%)	Median (%)	SD
CNN	89.5	96.9	93.4	93.5	1.53
QDA	88.0	94.5	91.2	91.4	1.42
RDF	86.2	93.5	90.0	89.8	1.63
LDA	80.9	90.2	86.8	87.1	1.68

### The results of the discriminant analyses

The Linear Discriminant Analysis (LDA) classified the seeds of *E*. *brachysperma* and *E*. *alsinastrum* with 98% and 96% accuracy, respectively. The first taxa (*E*. *brachysperma*) was confused with *E*. *triandra* in 2% of cases, while the seeds of the second (*E*. *alsinastrum*) were mistaken (at 4%) with *E*. *hexandra* (2%), *E*. *brochonii* (1%). and *E*. *macropoda* (1%) (Fig. 2A).

The least accuracy by LDA was observed for seeds of *E*. *californica* (55% correct classifications) and *E*. *campylosperma* (65%). The former was most commonly mistaken for *E*. *gussonei* (28%) and *E*. *hungarica* (17%), and the latter for *E*. *hungarica* (18%) and *E*. *hydropiper* (16%) (Fig. 2A). The LDA analysis most often incorrectly classified specimens of *E*. *alsinastrum*, *E*. *gussonei*, *E*. *hexandra*, *E*. *hungarica*, *E*. *macropoda*, *E*. *triandra* with other taxa (confused with three other taxa); however, in all cases, the rate of incorrect classification did not exceed 9% (Fig. 2A).

The Random Decision Forest (RDF) analysis recognised the seeds of *E*. *brachysperma*, *E*. *brochonii*, *E*. *orthosperma* and *E*. *triandra* with 97% accuracy. The first taxon (*E*. *brachysperma*) was confused with *E*. *triandra* in 3% of cases. The second taxon, *E*. *brochonii*, was mistaken for *E*. *alsinastrum* in 1% of cases, *E*. *brachysperma* in 1% and *E*. *triandra* in 1%. Finally, *E*. *orthosperma* was confusd with *E*. *alsinastrum*; while *E*. *triandra* was mistaken for *E*. *hexandra* in 2% of cases and *E*. *brachysperma* in 1% (Fig. 2B).

However, the least accuracy by RDF was noted for *E*. *californica* (69%). This taxon was most commonly mistaken for *E*. *hungarica* (in 16% of cases) and *E*. *gussonei* (14%) (Fig. 2B). It can also be seen that the RDF most often misclassified *E*. *gussonei* and *E*. *macropoda* (confused with four other taxa). The remaining seeds were on average confused with two or three species (Fig. 2B).

The Quadratic Discriminant Analysis (QDA) recognised the seeds of *E*. *brochonii* and *E*. *orthosperma* with 99% accuracy. The former (*E*. *brochonii*) was confused with *E*. *hexandra* in 1% of cases, and the latter (*E*. *orthosperma*) with *E*. *alsinastrum* in 1% (Fig. 2C). The least accuracy for the QDA was noted for *E*. *hexandra* (81% of correct classifications) and *E*. *californica* (71%). In the case of the former, it was mainly confused with *E*. *hungarica* (incorrect in 22% of cases) and *E*. *gussonei* (7%), while *E*. *hexandra* was incorrectly classified as *E*. *macropoda* (14%), *E*. *brochonii* (4%) and *E*. *alsinastrum* (1%) (Fig. 2C). The Quadratic Discriminant Analysis most frequently misclassified *E*. *gussonei* and *E*. *macropoda* (confused with four other taxa). The remaining seeds were on average confused with more than two species (Fig. 2C).

In the QDA method, five taxa had an average match of at least 95%, while in RDF and LDA, four. All methods demonstrated the least accuracy for *E*. *californica* (LDA 55%, RDF 69% and QDA 71%), being most often confused with *E*. *gussonei* and *E*. *hungarica*: the respective misclassification rates were 28% and 17% in LDA, 14% and 16% in RDF, and 7% and 22% for QDA. For RDF and QDA, an average level of fit (about 80% to 90%) was noted for *E*. *hexandra*, *E*. *hungarica*, *E*. *macropoda* and *E*. *campylosperma*, with the highest matches (i.e. over 90%) recorded for *E*. *alsinastrum*, *E*. *brachysperma*, *E*. *brochonii*, *E*. *gussonei*, *E*. *hungarica*, *E*. *hydropiper*, *E*. *orthosperma* and *E*. *triandra*.

In the LDA and RDF models, among all the features studied, the characteristics that had the greatest impact on the prediction process were: angle (44% and 31% respectively), pits (31%, 33%) and rectangle a (41% and 19% respectively) (Supporting Information Fig. 9A,B). In contrast, the least impact was observed for profile (8%) and surface (20%) for LDA, and surface (5%) and profile (6%) for RDA analysis. In the case of QDA, the greatest impact was noted for rectangle b, rectangle a and surface (48%, 45% and 45% respectively), and the least for profile (27%) and pits (36%) (Supporting Information Fig. 9C).

### CNN networks analysis

The CNN analysis classified *E*. *brochonii* and *E*. *orthosperma* with the highest accuracy (100%), followed by *E*. *alsinastrum* and *E*. *trianda* (96% accuracy), and *E*. *californica* and *E*. *hungarica* (95% accuracy). Finally, *E*. *brachysperma* and *E*. *hydropiper* were recognized with 94% precision (Fig. 2D). On the other hand, *E*. *macropoda* and *E*. *hexandra* were classified similarly or slightly worse than the classical method, with respective accuracy of 88% and 78%. In the case of the former, it was incorrectly classified as *E*. *macropoda* (9%), *E*. *brachysperma* (5%), *E*. *alsinastrum* (4%), *E*. *hungarica* (2%) and *E*. *triandra* (2% error), while the latter was conused with *E*. *hexandra* (8%), *E*. *alsinastrum* (2%) and *E*. *orthosperma* (2% error) (Fig. 2D).

In addition, the CNN network analysis most often incorrectly classified *E*. *hexandra* and *E*. *hungarica* (with five species) as well as *E*. *gussonei* and *E*. *macropoda* (with three taxa). The remaining seeds were typically mistaken for two or one species (Fig. 2D).

The results obtained are confirmed by the ROC curve and the AUC value. The CNN method is better than the other used classical statistical methods based on morphometry. All methods obtained a high AUC value, close to 1. Thus, it should be considered that, according to the result obtained, the differences between the various machine learning results are good. However, this does not change the fact that the best results and AUC values are obtained using deep machine learning CNN (Fig. 3).

## Discussion

Our findings indicated that CNN-based deep machine learning offerered similar or better quality predictions than the LDA, RDF and QDA methods in 10 cases.

In the CNN analysis, the seeds of *E*. *brochonii* and *E*. *orthosperma* were recognized with an accuracy of 100%, and the remaining taxa, apart from *E*. *hexandra* and *E*. *macropoda*, were recognized with over 90% precision. Our findings are consistent with the those of morphological studies: the mentioned species are clearly distinct from the others by the length and width of their seeds^2,36^.

The CNN analysis demonstrated the least accuracy for the *E*. *hexandra* and *E*. *macropoda* seeds (78% and 88%, respectively). This is in line with Popiela et al.^16^, who note that the seeds of these species can be easily confused, especially if only a few are assessed. They also found these taxa to demonstrate much greater seed variability than other taxa of the described genus; this has also been confirmed elsewhere^15,16,37^.

The QDA, LDA and RDF analyses, based on the human- measured parameters demonstrated a lower percentage of correct classifications (mean of correct classifications in the range of 86–91%) than the CNN analysis (mean 93%). The QDA method demonstrated a mean match at least 95% for six taxa, RDF for five taxa and LDA for four.

The highest matches (over 90%) were demonstrated for *E*. *brochonii*, *E*. *triandra*, *E*. *orthosperma*, *E*. *hydropiper*, *E*. *brachysperma*, *E*. *alsinastrum* and *E*. *gussonei*. In all methods, the worst classified species was *E*. *californica* (55% LDA; 71% RDF and QDA), which was most often confused with *E*. *hungarica*; this was confused in 16% of cases by LDA, 17% of cases by RDF, and as much as 22% by QDA. The variability of the tested *Elatine* seeds is mainly determined by characteristics related to their size, especially their area and circumference^2,9,11–14^.

The results of the QDA, LDA, RDF analyzes indicated that the most taxonomically useful features are the angle of the seed curvature and the number of pits in the seed coat, with *rectangle “a”* having a lesser influence; they also demonstrate that extensive variation exists both between species and populations within a species. These findings are in line with those of Molnár et al.^2^, Sramkó et al.^9^, Uotila^12,36^, Misfud^15^ and Popiela et al.^16^.

However, Misfud^15^ highlights the taxonomic importance of the number and shape of pits in the seed coat in populations of *E*. *gussonei* and *E*. *macropoda* from Malta and Mallorca. Similarly, Molnár et al.^2^, Molnár, Popiela and Lukács^3^, and Popiela et al.^16^ propose that the shape and number of pits can also be used to distinguish between the seeds of individual species. Our results indicate that this feature can be omitted because we do not need to report it separately when using CNN analysis. The differences observed in the constancy of features, and hence their variation within a taxon, may be due to the unclear taxonomic status and phylogenetic relationships between some of the analyzed species^8,9,38^. *Elatine gussonei* was first described as a variety of *E*. *hydropiper* (*E. hydropiper* var. *gussonei* Sommier); however, this taxon was later classified as a separate species^38^ and remains so today^2,3,15,39^. *Elatine campylosperma* was described by Seubert^40^ from Sardinia; although it was later synonymous with *E*. *macropoda*^11,35,41^, it is now recognized as a separate species, and the only known diploid (2n = 18) species in the *Elatine* genus^7,9^. Finally, *Elatine hungarica* was last collected from a site in Hungary in 1960 but later rediscovered in this area in 1998^42^; its taxonomic status has been discussed over the years before being recognized as a species^2^.

*Elatine hexandra* is likely of hybrid origin (2n = 108), and its geographical range coincides with that of *E*. *brochonii* and other species from the subsection *Macropodae* Sramkó A. Molnár & Popiela, in the Mediterranean Basin^9^. In addition, Razifard^10^ emphasize the recent origin of *E*. *brachysperma*.

Interestingly, the results of phylogenetic studies indicate that the main seed shapes (straight/almost straight, curved, U-shaped) are not associated with monophyletic clades. The simple seed shape appears in both older and younger lines, suggesting that it may have arisen many times during the evolution of the genus^9^.

## Supplementary Information

Supplementary Information.

## Data Availability

All data generated or analysed during this study are included in this published article and its supplementary information files.
