# Supplementary material for: Comparison of discriminant methods and deep learning analysis in plant taxonomy: a case study of Elatine
Source: Sci Rep. 2022 Nov 28;12:20450. doi: 10.1038/s41598-022-24660-1 (PMC9705712; doi:10.1038/s41598-022-24660-1)
Supplement: Supplementary file 1 — Supplementary Information. [file 41598_2022_24660_MOESM1_ESM.pdf]

Supplementary Information

Sample photos of seeds

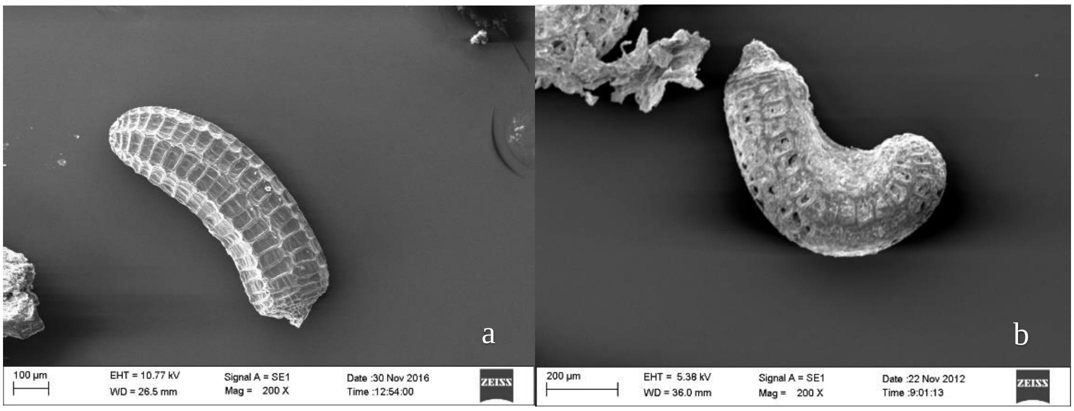

Fig. 1. Exemplary input images acquired from an electron microscope; a – *Elatine alsinastrum*; b – *Elatine gussonei*.

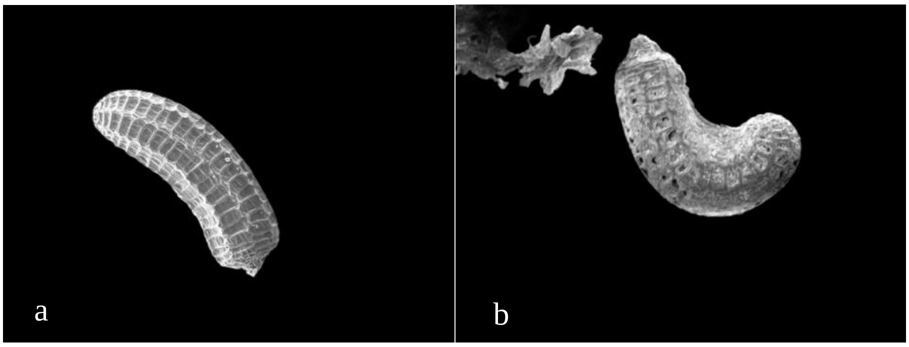

Fig. 2. An example where seeds were extracted from the surrounding background for further CNN analysis; a – *Elatine alsinastrum*; b – *Elatine gussonei*.

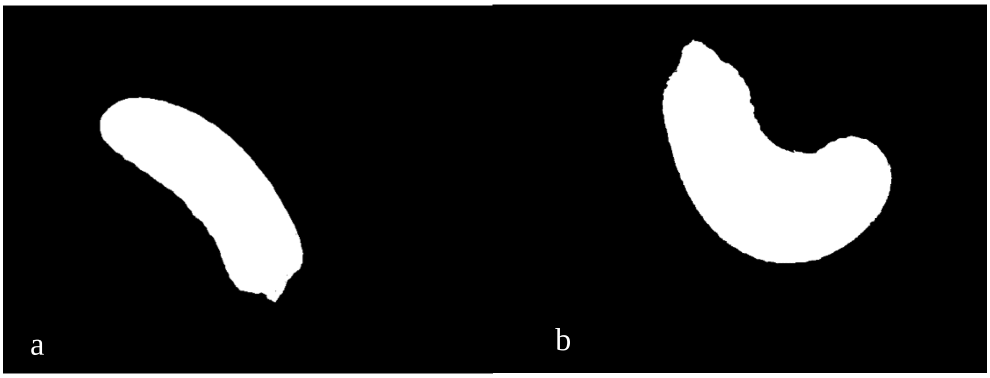

Fig. 3. The result of the binarization process used to select objects for CNN analysis; a – *Elatine alsinastrum;* b – *Elatine gussonei*.

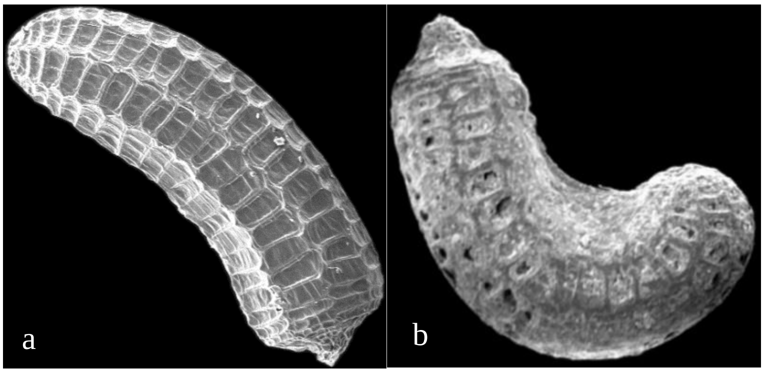

Fig. 4. Prepared images for CNN deep machine learning analysis; a – *Elatine alsinastrum*; b – *Elatine gussonei*.

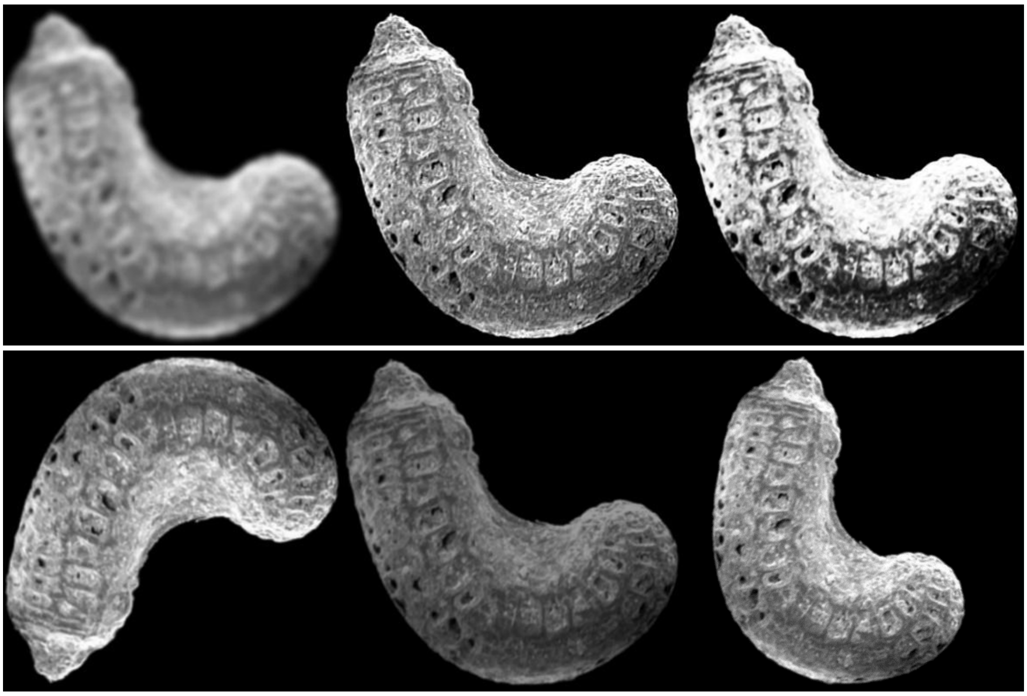

Fig. 5. Examples of the classical data transformation processes used for augmentation. *Elatine gussonei*.

The structure of the net used for image classification

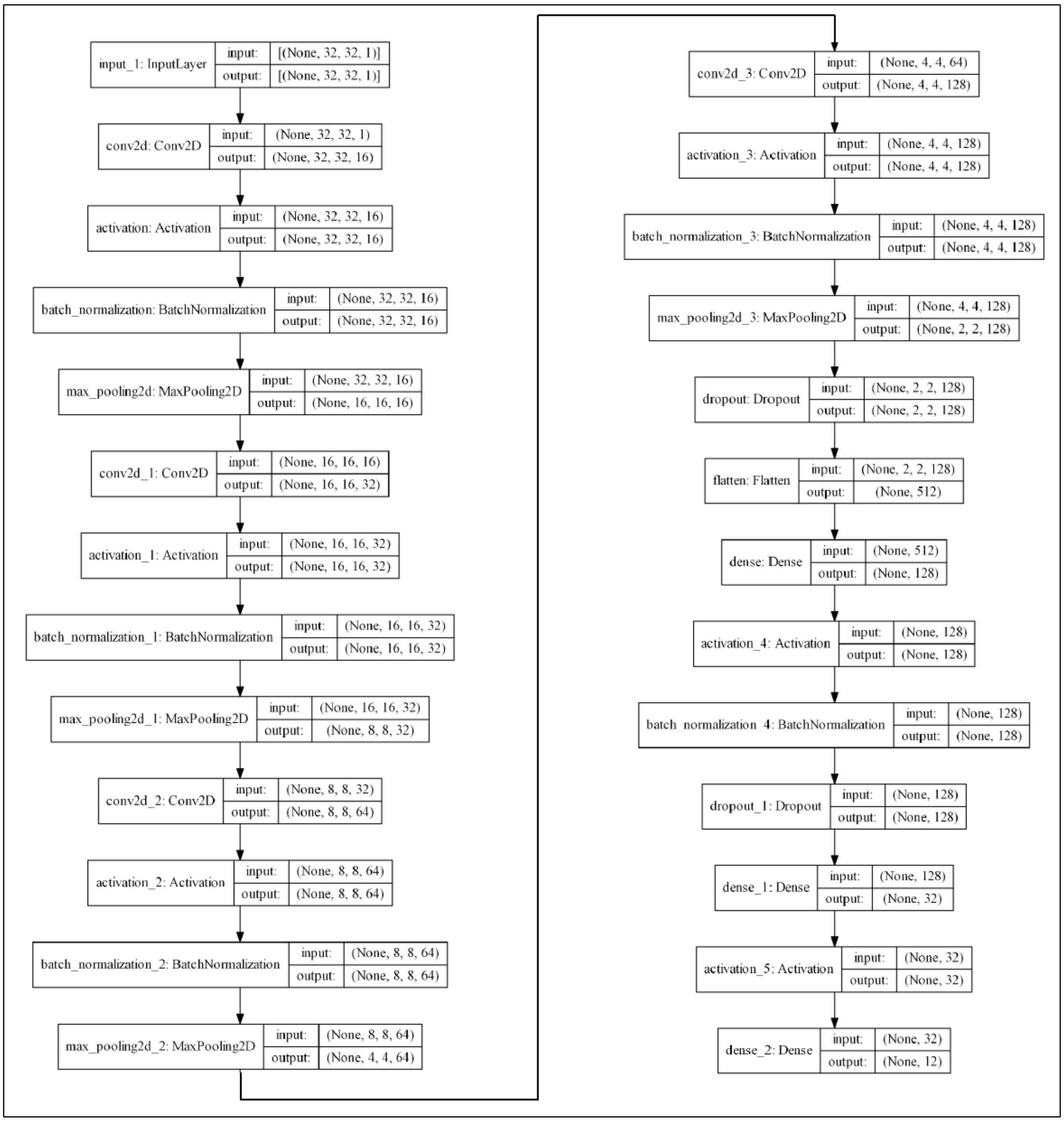

Fig. 6. The structure of the net used for image classification.

Selected morphological features for statistical analyses

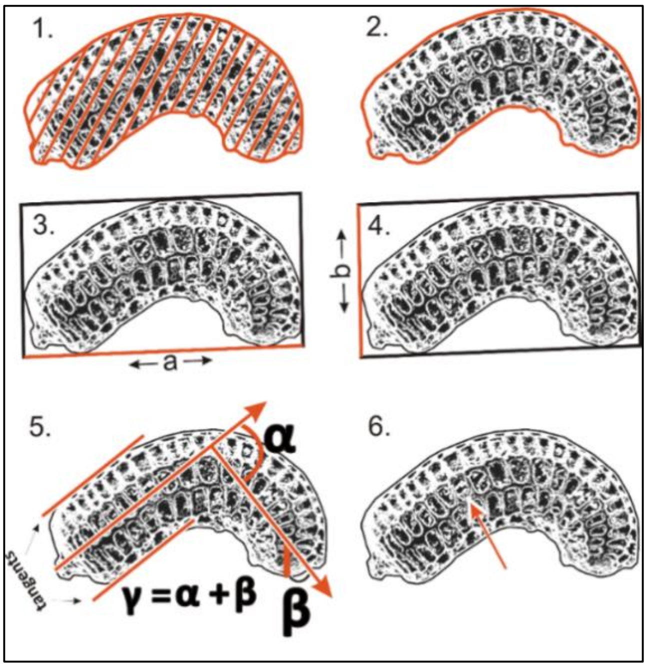

Fig. 7. Selected morphological features for statistical analyses: 1. – surface, 2. – profile, 3. – rectangle a, 4. – rectangle b, 5. – the angle of curvature, 6. – number of pits in the middle row.

Distribution of the results for RDF, LDA, QDA and CNN analysis

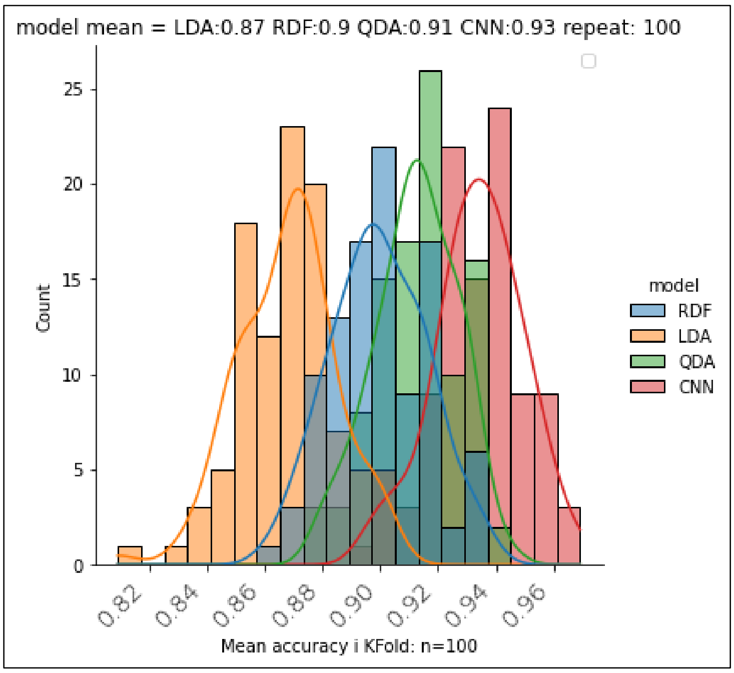

Fig. 8. Distribution of the fit results (accuracy) following 100 repetitions using RDF, LDA, QDA and CNN.

Permutation significance of the selected variables for LDA, RDF and QDA analysis.

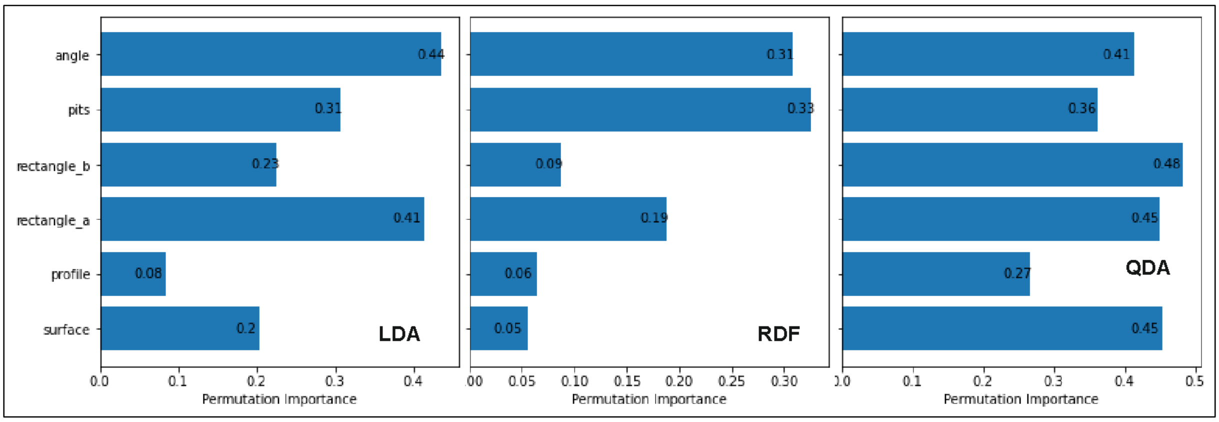

Fig. 9. Permutation significance of the selected variables for individual analyses: A – LDA; B – RDF and C – QDA analysis.
